# Women’s perspectives on the management and consequences of hyperemesis gravidarum – a descriptive interview study

**DOI:** 10.1080/02813432.2019.1569424

**Published:** 2019-03-01

**Authors:** Gro C. Havnen, Maria Bich-Thuy Truong, Mai-Linh H. Do, Kristine Heitmann, Lone Holst, Hedvig Nordeng

**Affiliations:** a Regional Medicines Information and Pharmacovigilance Centre (RELIS), Oslo University Hospital, Oslo, Norway;; b PharmacoEpidemiology and Drug Safety Research Group, Department of Pharmacy, University of Oslo, Oslo, Norway;; c Regional Medicines Information and Pharmacovigilance Centre (RELIS), Haukeland University Hospital, Bergen, Norway;; d Department of Global Public Health and Primary Care, University of Bergen, Bergen, Norway;; e Department of Child Health and Development, Norwegian Institute of Public Health, Oslo, Norway

**Keywords:** Hyperemesis gravidarum (HG), Nausea and vomiting in pregnancy (NVP), patient perspectives, HG management, pregnancy

## Abstract

**Objective:** Hyperemesis gravidarum (HG) affects 0.3–3% of pregnant women and is a leading cause of hospitalization in early pregnancy. The aim of the study was to investigate women’s treatment and management of HG, as well as the consequences of HG on women’s daily life.

**Design and setting:** A cross-sectional study based on a structured telephone interview and an online questionnaire. Participants were recruited by social media and by the Norwegian patient’s organization for HG.

**Subjects:** Norwegian women that experienced HG.

**Main outcome measure:** Women’s perspectives on management and consequences of HG.

**Results:** The study included 107 women. Maternal morbidity was profound; about 3/4 of participants were hospitalized due to HG, and the majority showed clinical signs of dehydration (79%), ketonuria (75%), and >5% weight loss (84%). Antiemetics were used by >90% and frequently prescribed “as needed”. Metoclopramide (71%) and meclozine (51%) were most commonly used. Participants described HG as having severe psychosocial consequences and profound impact on daily activities. Almost two out of five reported thoughts of elective abortion, and 8 women had at least one elective pregnancy termination due to HG. Overall, 20 women (19%) changed GPs due to dissatisfaction with HG management.

**Conclusion:** Despite the high psychosocial burden and major impact on daily activities, many women with HG reported a lack of support from healthcare professionals and suboptimal management. Greater awareness and knowledge among healthcare professionals is needed to improve care for women with HG.

**Key Points**
There is a paucity of studies on management and the consequences of HG on women’s daily lives and psychosocial burden. We found that:
• Many women described HG as one of their worst life experiences with profound morbidity.
• Many women reported suboptimal management of HG and lack of support from healthcare professionals.
• Greater understanding of patient perspectives among healthcare professionals is important to improve care and management for HG patients.

## Introduction

Hyperemesis gravidarum (HG) is considered the most severe manifestation of nausea and vomiting in pregnancy (NVP) and occurs in 0.3–3% of all pregnancies [1,2]. HG causes weight loss, dehydration, electrolyte disturbances, and nutritional deficiency. HG has also been associated with premature delivery and small-for-gestational-age infants [3]. Despite an apparently relatively low incidence, the condition is the most common cause of early pregnancy hospital admissions [4]. Moreover, recurrence in consecutive pregnancies is high, ranging from 15% to over 80% [5,6].

Despite the severity of HG, there is no universal consensus on the definition of the condition. Therefore, HG is a clinical diagnosis without uniform criteria [7,8]. Common criteria for diagnosis are persistent vomiting accompanied by weight loss exceeding 5% of pre-pregnancy body weight, ketonuria, and dehydration, which in many cases, requires intravenous rehydration. The aetiology of HG is not clearly understood, but it is thought to be multifactorial and complex [7,9]. Recent studies have also suggested that some women are more at risk due to genetic factors [7]. Because there is no clear aetiology for HG, treatment focuses on reducing the severity of symptoms and preventing further complications such as electrolyte disturbances and malnutrition. First generation antihistamines are considered safe and the first-line antiemetic treatment. Dopamine receptor antagonists, like metoclopramide and phenothiazines, are considered second-line drug therapies. Ondansetron is used after other antiemetic medicines have failed [10–12]. In several countries, a combination of doxylamine and vitamin B6 (pyridoxine) has been licensed for treating NVP [13–15]. However, no medicines have been licensed for treating NVP or HG in Scandinavia.

Most research related to NVP and HG has focused on aetiology and treatment options, rather than the patients’ experiences of HG management and consequences. However, several studies have documented that NVP and HG can have a profound negative impact on women’s daily life functions; it negatively affects the quality of life, daily activities, social functions, work capacities/abilities, partner and family relationships, and parenting [16–20]. A recent systematic review and meta-analysis also found that women with HG ran significantly elevated risks of depression and anxiety [21]. The condition can be so debilitating and impose so much distress that women with severe NVP and HG have considered or chosen to terminate an otherwise wanted pregnancy [22,23]. In a web-based survey of 808 women affected by HG, 15% of respondents reported terminating at least one pregnancy, due to HG [23]. In the same survey, 37% of respondents reported that they would not consider or plan any more pregnancies, which further illustrated the negative effect of HG on reproduction [20]. Christodoulou-Smith *et al.* performed an online survey that included 377 women with HG and 233 controls. They found that as many as 18% of women with HG fulfilled the criteria of posttraumatic stress syndrome [24]. In that study, women with HG were also more likely to have experienced negative postpartum life events, including financial, marital, career, and psychiatric problems, compared to the control group of women [24].

The perception that NVP is a natural part of pregnancy, which poses only minor discomfort, may imply that the symptoms are trivialized, both by healthcare professionals and the public. This perception could lead to lack of support, under-diagnosis, and under-treatment of women with severe NVP. There is a paucity of studies on management and the consequences of HG on women’s daily lives and psychosocial burden. Moreover, to the best of our knowledge, no study has investigated women’s experiences of HG in a Scandinavian country using a structured interview to capture women’s management of HG and impact on daily life and functioning. The main objective of this study was to investigate patients’ pharmacological treatment and management of HG. We also aimed to gain knowledge about the consequences of HG. These results may be of great relevance for general practitioners and all other healthcare professionals who encounter pregnant women in antenatal care.

## Material and methods

### Design, recruitment and data collection

This study had a cross-sectional design. Data was collected through a structured telephone interview and a structured online questionnaire. The interview guide and online questionnaire were developed based on results from previous studies [17,20,25] and in collaboration with the Norwegian patients's organization for HG.

Due to the relative rarity of the disease, limited inclusion criteria were applied. All women (age ≥ 18 years) that had experienced HG during their current or former pregnancy were invited to participate in the study. An invitation to participate in the study was posted on several social media platforms including a study Facebook page between mid-January and the end of February 2015. Information about the study was also disseminated by the Norwegian patient's organization for HG to its members. All women that had signed an electronic declaration of consent and provided contact information were contacted and invited to participate in a telephone interview.

The interview included both closed-ended and open-ended questions to capture women’s experiences and perceptions, and the online questionnaire included closed-ended questions with multiple choice and ranking. The main topics in the interview and online questionnaire were: (*i*) HG symptoms and severity, (*ii*) pharmacological treatment and management of HG, and (*iii*) psychosocial consequences and impact on daily activities. To limit the duration of the telephone interviews, questions more suitable for self-completion were delivered in a self-administrated online questionnaire (www.questback.com).

The structured telephone interview was conducted by a specially trained pharmacist/nurse. Participants with more than one HG pregnancy were instructed to select a specific HG pregnancy that they thought would be most interesting to healthcare professionals that care for patients with HG (hereby referred to as the selected pregnancy). The women were asked to respond to questions with the selected HG pregnancy in mind.

#### Diagnostic criteria of HG

HG was defined as either a medical diagnosis of HG or at least two out of three clinical features of HG: (1) weight loss exceeding 5% of the pre-pregnancy weight; and/or (2) ketones on a urine analysis; and/or (3) dehydration and/or an electrolyte imbalance. We excluded women who did not fulfill these diagnostic criteria and women who had experienced HG more than 2 years prior to the study.

### Measures

#### Maternal characteristics

Socio-demographic characteristics were collected in the online questionnaire (maternal age, marital status, education, and occupation). Information on a variety of maternal characteristics was obtained during the interview; e.g. parity, number of pregnancies with HG, risk factors, and time since the selected HG pregnancy. Pre-existing conditions/comorbidity was collected in the online questionnaire.

#### HG symptoms and severity

During the interview, women were asked several detailed questions about the time course and severity of HG symptoms, including weight loss, ketonuria, dehydration and/or electrolyte imbalance, and hospital admissions, due to HG.

#### Pharmacological treatment and management

A range of questions about HG treatment were presented, including: use of antiemetic medications or other therapy for HG, e.g. complementary and alternative therapy (CAM); treatment of reflux comorbidity; the effectiveness of HG treatment (classified as none, moderate, or good); medication adherence; and treatment in the primary and/or secondary healthcare system.

Several questions about healthcare professionals’ attitudes and support were raised. For example, we asked whether women felt that they had been taken seriously; their satisfaction with the medical treatment provided; trust in their healthcare professionals; and whether they felt optimally cared for and understood by healthcare professionals. Moreover, each woman was asked about the level of knowledge her healthcare professionals had about HG. Answers were categorized as presented in Appendix Table 2. Women were also asked an open-ended question about what advice they would give healthcare professionals, based on their own HG experience.

**Table 1.** Hyperemesis gravidarum (HG) characteristics of the study participants.

HG characteristics	Number of patients; total *n* = 107
	*n* (%)
*HG clinical criteria*	
Ketonuria	80 (74.8)
Dehydration	85 (79.4)
≥5% weight loss	90 (84.1)
Two or three HG clinical criteria	90 (84.1)
All three HG clinical criteria	63 (58.9)
HG diagnosis, unspecified clinical symptoms	5 (4.7)
*Admitted to the hospital due to HG*	
Yes	77 (72.0)
No	29 (27.1)
Missing data	1 (0.9)
*Number of hospitalizations due to HG*	
0	29 (27.1)
1	37 (34.6)
2	25 (23.4)
>2	15 (14.0)
Missing data	1 (0.9)
Range of hospitalizations per patient	0–6
*Management of HG*	
Pharmacotherapy^a^	97 (90.7)
CAM^b^	94 (87.9)
*Number of pregnancies with HG*	
Only one	50 (46.7)
More than one	57 (53.3)
*Family members with HG*	
Yes	29 (27.1)
No	78 (72.9)

Data obtained from the interview.

^a^Includes meclozine, metoclopramide, ondansetron, prochlorperazine, or chlorpromazine, which were specifically ask for.

^b^CAM: complementary and alternative medicines, including acupuncture, acupressure, ginger, peppermint, and/or homeopathy.

**Table 2.** Pharmacologic treatment of hyperemesis gravidarum and effects.

	Number of patients; Total = 107	Treatment initiation, GW	Efficacy		
			None	Moderate	Good
	*n* (%)^a^	Median ± SD	*n* (%)	*n* (%)	*n* (%)
Metoclopramide	76 (71.0)	8.0 ± 2.8			
Continuous	31 (40.8)		8 (25.8)	4 (12.9)	19 (61.3)
As needed	43 (56.6)		21 (48.8)	4 (9.3)	18 (41.9)
Meclozine	55 (51.4)	7.0 ± 3.4			
Continuous	26 (47.3)		9 (34.6)	5 (19.2)	12 (46.2)
As needed	29 (52.7)		22 (75.9)	1 (3.4)	6 (20.7)
Ondansetron	31 (29.0)	12.0 ± 5.0			
Continuous	14 (45.2)		1 (7.1)	1 (7.1)	12 (85.7)
As needed	14 (45.2)		4 (28.6)	2 (14.3)	8 (57.1)
Promethazine	28 (26.2)	10.0 ± 5.3			
Continuous	9 (32.1)		2 (22.2)	5 (55.6)	2 (22.2)
As needed	17 (60.7)		9 (52.9)	6 (35.3)	2 (11.8)
Prochlorperazine	12 (11.2)	9.5 ± 2.9			
Continuous	4 (33.3)		0 (0.0)	2 (50.0)	2 (50.0)
As needed	7 (58.3)		4 (57.1)	2 (28.6)	1 (14.3)
Chlorpromazine	11 (10.3)	9.0 ± 7.6			
Continuous	2 (18.2)		0 (0.0)	1 (50.0)	1 (50.0)
As needed	6 (54.5)		2 (33.3)	3 (50.0)	1 (16.7)

Data obtained from the interview. GW: gestational week; SD: standard deviation.

^a^Total numbers do not add up for the resepective medications, due to missing values for metoclopramide (*n* = 2), ondansetron (*n* = 3), promethazine (*n* = 2), prochlorperazine (*n* = 1), and chlorpromazine (*n* = 3).

#### Impact on daily activities and psychosocial consequences

Information was collected regarding the consequences and impact of HG on emotional well-being, social functioning, daily activities, and sick leave. Each woman was specifically asked about whether HG had an impact on her plan for future pregnancies and whether she had thoughts about or carried out an elective abortion due to HG. The women were also asked to score their general well-being during the selected pregnancy on a scale from zero (the worst possible imaginable) to ten (as good as she had felt before the start of pregnancy). The remaining data were categorized as presented in Table 3.

**Table 3.** Negative impacts of hyperemesis gravidarum on daily activities and psychosocial burden on the study participants while experiencing HG.

Psychosocial burden	Number of women, *n* (%)	Daily activities	Number of women, *n* (%)
*Feeling depressed*^a^		*Performing household chores*^a^	
Always/most of the time/often	80 (83.3)	No impact	0 (0.0)
Sometimes	12 (12.5)	Minor impact	0 (0.0)
Rarely/never	4 (4.2)	Major impact	96 (100.0)
*Reduced quality of life*^a^		*Social life*^a^	
Always/often	90 (93.8)	No impact	0 (0.0)
Sometimes	6 (6.3)	Minor impact	0 (0.0)
Rarely/never	0 (0.0)	Major impact	96 (100.0)
*Thoughts of elective pregnancy termination*^b^		*Relationship with partner*^a^	
Yes, always/often	12 (11.2)	No impact	16 (17.4)
Yes, sometimes	24 (22.4)	Minor impact	32 (34.8)
Yes, non-specified	5 (4.7)	Major impact	44 (47.8)
Never	66 (61.7)		
*Considering future pregnancies after HG pregnancy*^b^		*Ability to care for children*^a^	
Yes	48 (44.9)	No impact	0 (0.0)
No, due to HG	35 (32.7)	Minor impact	4 (7.3)
No, due to other reasons	11 (10.3)	Major impact	51 (92.7)
Unsure	13 (12.1)		
*General well-being*^b,c^		*Work capacity*^a^	
0	39 (36.4)	No impact	0 (0.0)
1	24 (22.4)	Minor impact	0 (0.0)
2–3	35 (32.7)	Major impact	89 (100.0)
≥4	9 (8.4)		

Data obtained from the interview.

^a^Question from the online questionnaire, total *n* = 96. Numbers do not always add up to 96, because the questions were not relevant for women without a partner (*n* = 4), without children (*n* = 41), or without work (*n* = 7).

^b^Questions from interview, *n* = 107.

^c^General well-being rated from 0 (= worst possible) to 10 (= as good as pre-pregnancy).

### Data analysis and extraction

Descriptive statistics were utilized as appropriate. Continuous variables were compared with the non-parametric Wilcoxon rank-sum test. A p-value of less than 0.05 was considered significant. All statistical analyses were performed with the Data Analysis and Statistical Software, Stata/MP version 14.

Data extracting of the open-ended questions and the selection of representative statements in Table 4 was done by three of the authors (GCH, BT, MLD).

**Table 4.** A selection of representative quotations from women with HG obtained during the study interviews.

Trivialisation	The physician did not take me seriously. I dreaded going there, every time. He told me that pregnancy is not a disease and that there was no such thing as hyperemesis gravidarum. I felt insignificant.*Woman with 18% weight loss*
	They all thought that I was exaggerating: psychologists, friends, and family. Nobody understood me. *Woman hospitalized for 5 days, 5% weight lost*
	I felt that I did finally get help, but it took so long. I told my caregivers that I was not capable of eating and that I lost weight, but no one took me seriously. *Woman hospitalized for 4 days, 8% weight loss*
	My midwife believed that HG was nonsense. *Women hospitalized for 5 days, 8% weight loss*
	The attending physician at the hospital did not understand how it was to have HG. She told me to eat and that I didn’t need to be hospitalized. I was hurt and felt stupid. *Woman hospitalized for 2 days, 13% weight loss*
Medication	I did not receive any treatment. I was told not to take any anti-emetic medications. I did not get any advice other than to eat some crackers. *Woman hospitalized for 7 days, 18% weight loss*
	I was told to take metoclopramide as needed and no more than necessary. I was also told to pause treatment occasionally. *Woman hospitalized for 2 days, 7% weight loss*
Severity	I wanted to die, there and then. Absolutely awful. I would lie on the bathroom floor for hours. In the beginning, I tried to stay hydrated, but I could not retain anything. I was absolutely sensitive to all sound and light. Every move could aggravate the nausea. *Woman hospitalized for 12 days, 14% weight loss*
	I considered an elective abortion, even though this was a wanted pregnancy that we had planned for a long time. *Women hospitalized for 72 days, 16% weight loss*
Healthcare	The physician never told me that I had a HG diagnosis. I found out about HG later on, via the Facebook forum. The physician wrote that I had “extreme nausea in pregnancy”, but never explained to me what it really was. *Woman hospitalized for 25 days, 7% weight loss*
	I have been in and out of the hospital/emergency room several times. Some physicians were clear about my HG condition, other physicians told me that I was mentally ill. I switched midwife because she told me that I would be fine after week 10. *Woman hospitalized for 30 days, 10% weight loss*
Consequences	HG was awful. I wasn’t myself, and I felt guilty because I couldn’t care for my family. It ruined our relationship. I was socially disabled. *Woman with 6% weight loss*
	I got a new job 3 months before the pregnancy. My co-workers didn’t understand. HG has weakened me. I’m now depressed and still on sick leave (after birth). I had to quit my job and move to another place. *Women hospitalized for 7 days, 14% weight loss*

### Ethics

The study was approved by the Regional Committee for Medical and Health Research Ethics, Region West, in Norway (2014/1065/REK Vest). All data were handled confidentially and stored anonymously. Contact information and study data were stored separately. The contact information was deleted after data collection was completed.

## Results

**Appendix Figure 1.**
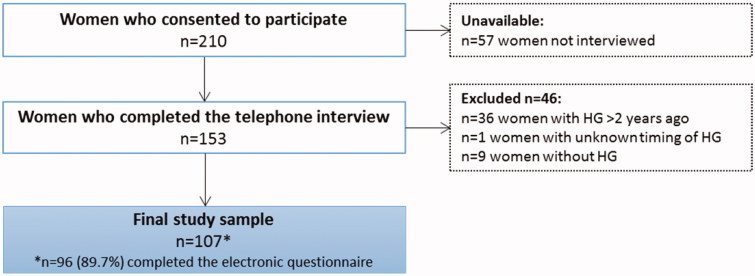
Flowchart of women that met the inclusion and exclusion criteria for the final study sample. HG: hyperemesis gravidarum. *Of the 107 women included, 96 (89.7%) completed the online questionnaire.

A total of 210 women consented to participate in the study, and 153 interviews were performed. The interviews lasted an average of 48 minutes, ranging from 25 to 120 minutes. Of these, 107 women met the inclusion criteria and were included in the final study sample, and 96 women (89.7%) completed the online questionnaire. A flowchart outlining the patient inclusions and exclusions that led to the final study sample is depicted in Appendix Figure 1. The average age of study participants was 30.5 ± 4.5 years. Participants were mostly married, cohabiting, or in a relationship (92/96, 95.8%), had a university or college education (60/96, 62.5%), and were not pregnant (76/107, 71.0%) at the time of study inclusion (Appendix Table 1). Nearly 3 out of 4 women (77/107, 72.0%) had experienced hyperemesis gravidarum (HG) during the year prior to the study. Thirteen women with more than one HG pregnancy chose to focus on their first experience with HG.

### HG symptoms and severity

Symptoms of nausea arose, on average, in gestational week (GW) 5 (mean GW: 5.2 ± 1.2 weeks), and the majority (*n* = 98/107, 91.6%) had experienced nausea by GW 6. Fifty-four women (54/105, 51.4%) experienced nausea throughout the entire pregnancy. The majority of these women (40/54, 74.1%), however, experienced improvement in the intensity of nausea as the pregnancy proceeded. Seventy-three women (73/96, 76.0%) reported that severe nausea symptoms persisted throughout the entire day (24 h), and 29/107 (27.1%) women could not eat or drink anything when HG symptoms were most intense. Women that lost weight due to HG (101/107, 94.4%), on average, lost 9.9% ± 5.5% (range: 0–25%) of their pre-pregnancy body weight. Seventy-seven (77/107, 72.0%) women were hospitalized due to HG for an average of 14 ± 18 days (median: 7, range: 1–105 days). Table 1 gives an overview of the HG characteristics in the study sample.

### Pharmacological treatment and management of HG

In total, 90.7% of women reported that they had used at least one antiemetic medication. The most common medications were metoclopramide (71.0%) and meclozine (51.4%) (Table 2). Symptoms of nausea arose early in pregnancy, but pharmacological treatment was generally initiated later in pregnancy (Table 2). Treatment durations were significantly shorter among women that reported no efficacy compared to women that reported good efficacy (metoclopramide: median 4 vs. 56 days, z = −6.00, *p* < .001; meclozine: median 7 vs. 56 days, z = 4.95, *p* < .001; and prochlorperazine: median 2 vs. 22 days, z = −2.14, *p* = .032).

The interview revealed that the women felt confused about medication regimens. Several women reported that they discontinued treatment after using a few tablets without efficacy, and 11.4% of participants (12/105) did not adhere to the physicians’ prescription. Specific reasons for poor adherence were a perceived fear of teratogenicity and fear after reading the patient information leaflet.

In addition to medications, 87.9% (94/107) of women used one or more CAMs. Of these, only 12.8% (12/94) reported a positive effect on HG. Heartburn and acid reflux were common (73/96, 76.0%) among the study participants. However, only 24.7% (18/73) used medications for these indications. All women that received intravenous fluid therapy (*n* = 81) reported that it had a positive effect on HG. Two women received gastric tube feeding after admission to the hospital.

In the interviews, 22 of 107 (20.6%) women reported that they were dissatisfied with their pharmacological treatment for HG. The remaining women (85/107, 79.4%) were satisfied to varying degrees. Twenty women (18.7%) changed their GPs because they were not satisfied with the treatment they had received for HG. Fifty-five women (51.4%) and 42 women (51.2%) felt that their GPs and midwives respectively had no knowledge about HG. The study participants felt, in general, better cared for and understood by hospital physicians than with their GPs (Appendix Table 2).

Thirty-eight women (35.5%) said specifically that they were afraid that they would not be believed or taken seriously before their first contact with the healthcare system. Nearly half of participants (48/107, 44.9%) reported that they experienced this after their first appointment. Many women also reported insensitive, or even hurtful, remarks from their GPs (Table 4). When women were asked to give advice to healthcare professional on how to improve care for patients with HG, the majority stated “Take us seriously” (71/107, 66.4%).

### Impact on daily activities and psychosocial consequences

All participants reported that HG had had a major impact on their daily activities and sociability (Table 3). Several women expressed severe distress and functional disability caused by HG. In total, 8/107 (7.5%) women reported a history of elective pregnancy termination due to HG. The experience with HG also had a profound impact on women’s thoughts about whether she wanted another child in the future (Table 3).

Most women (*n* = 101) had been on sick leave during pregnancy due to HG. Among these, 46.5% (47/101) were absent from work throughout the entire pregnancy, 28.7% (29/101) were absent from work for an average of 18.9 ± 5.5 weeks, and the remaining (25/101, 24.8%) were currently on sick leave or did not specify sick leave duration. Notably, 55/101 women (54.5%) had not received any pharmacological treatment for HG before being prescribed sick leave.

The participants described HG as one of the worst life experiences they had ever had. They used expressions like; “inhuman torture”, “like being nine months in prison”, “poor self-esteem”, and “the nausea was much worse than giving birth, I would rather give birth 10 times instead of having HG”. Selected quotations from women about their experiences with HG and their satisfaction with the healthcare system are shown in Table 4.

## Discussion

Although a relatively rare condition, HG is a leading cause of maternal hospitalization during early pregnancy. This study revealed several findings of clinical importance. A striking finding was that the women describe HG as one of their worst life experiences with profound morbidity and severe psychosocial consequences. Despite this troubling finding, about half of the women felt their healthcare professionals had no knowledge about the condition. Many women also reported insensitive or even hurtful remarks from their GPs. As a result of poor experiences, many women feared the possibility of HG in a future pregnancy, and due to this fear, several study participants did not wish to become pregnant again. This finding implied that HG had far-reaching consequences for the woman and her family.

In contrast to NVP, which typically resolves by the end of the first trimester or during the second trimester [26,27], half of the women with HG in our study experienced nausea and vomiting throughout the entire pregnancy, which added extra strain for these women. Women’s negative experiences were prominent at the first consultation with the GPs, where nearly half of the women reported that they were not believed or taken seriously. In our study, nearly 20% of participants chose to change GPs, due to unsatisfactory HG management; in comparison to the general Norwegian population where about 7.5% switch GPs in 2016 [28]. The majority of women stated that they wished that healthcare professionals would take them more seriously. Other studies have also reported that patients with HG commonly wished for empathy, but frequently reported a lack of support from healthcare professionals and the feeling that they represented a burden [20,25].

We acknowledge the challenging situation GPs are in, especially since there are few high-quality studies assessing the effectiveness of antiemetics for HG [29,30], and that the illness may progress over time. However, our study showed that there is room for improvements in the use of antiemetics and HG management. Several authors have pointed out that early recognition and management of all grades of NVP can prevent delay in diagnosis of HG and reduce the likelihood of hospital admission [4,9,10]. Therefore, it is of concern that pharmacologic treatment among women in our study was generally initiated late, and that about half of the women were prescribed sick leave before receiving pharmacologic treatment. Typically, women were advised to use antiemetics, only when the symptoms became severe. Our study revealed that over half of the women took antiemetic medications as needed, and not regularly, on a daily basis, which has been recommended for the optimal treatment effect [31]. Indeed, in our study, a clear tendency of higher efficacy was observed when medications were taken continuously, compared to medications taken intermittently. Furthermore, an unexpected finding was that more women in our study used metoclopramide (71.0%) than meclozine (51.4%), the latter being the first-line choice of antiemetic in Norway. In addition, the EMA released in 2013 a warning recommending limiting use of metoclopramide to maximum 5 days to reduce the risk of neurological side effects [32]. This finding could indicate that the warning was not well known among healthcare professionals in Norway, or that the benefits of using metoclopramide were considered to outweigh a possible risk of extrapyramidal side effects in this severely affected patient group.

In 1997, Mazzotta et al. reported that both healthcare professionals and patients with HG were reluctant to use antiemetics, due to a perception that they carried teratogenic risk [33]. This was also discussed by Koren and Levichek in 2002 [34]. This perception might cause a delay in initiating pharmacotherapy. In prescribing medical treatment during pregnancy, one should always consider benefit versus risk trade-off for both the mother and the foetus. We found that, in extreme cases, women felt that the only option were termination of otherwise wanted pregnancies, due to the severity of their symptoms. This finding profoundly illustrates that withholding treatment could be fatal for the foetus. Disturbingly, about 2/5 of the women in our study had thoughts of elective pregnancy termination; moreover, some women reported a history of elective pregnancy termination, due to HG. Bearing these findings in mind, the risks of using antiemetic therapy are lower, by far, than the risks associated with the untreated condition.

In addition to the fear of teratogenicity, reluctance to prescribe and use of antiemetic therapy could be due to a lack of knowledge. This possibility was supported by the fact that, in our study, half the women felt that their GP had no knowledge about HG. However, the women’s perspectives of their healthcare providers’ knowledge about HG do not necessarily reflect their true knowledge. Our results further suggested that, once women were hospitalized, their chances improved of receiving the support and treatment they needed. In most countries, however, antenatal care is provided in primary care facilities, and GPs must make a referral for a woman to receive specialist or hospital treatment.

Based on our findings, several recommendations can be made that may significantly improve care for patients with HG. First, it is important for healthcare professionals to acknowledge that symptoms and consequences of NVP can vary dramatically from woman to woman, from mild NVP to HG. There are validated, easy-to-use tools available, like the 24-h pregnancy-unique quantification of emesis scoring system (PUQE-24) [35], which can aid both the assessment of NVP severity and the effectiveness of management. The PUQE-24 is validated in a Norwegian setting [36], and it is also incorporated in the Norwegian guideline for emesis and hyperemesis gravidarum [37]. We believe that continuous treatment and frequent monitoring of symptoms and clinical signs of HG should be considered standard care. Unfortunately, the 2018 version of the antenatal care guideline in Norway does neither address NVP nor HG. Second, pharmacotherapy should not be withheld or delayed for women with nausea that is so severe that it impacts her daily life. At the same time, it is important to discuss the risks and benefits of medications with women; indeed, this discussion could promote patient adherence. Third, healthcare professionals should assess not only on the severity of symptoms but also the impact on daily life. Understanding the burden of HG and patients’ perspectives is important; our results should inform NVP guidelines, NVP management, and future research.

The main strength of this study was the extent of information collected during the structured interviews. These personal interviews provided detailed information about several aspects of patient perspectives on HG that would not be attainable in a survey. Although this study was conducted in one country, we do not believe that the findings on HG management are limited to Norway alone. Our clinical impressions and discussions with healthcare professionals in other countries also revealed similar attitudes and experiences. Though several prior studies have studied management and consequences of NVP, we do believe it is essential to study HG in specific due to its severe nature and potentially serious consequences of mismanagement of HG on maternal health.

This study had several limitations that should be taken into consideration. The major limitation was the fact that most data were collected retrospectively after HG. Nearly 75% of participants had experienced HG during the prior year; however, for 27 women, data were collected between 1 year and 2 years after the pregnancy. These long intervals might have had an impact on participant recall. Especially, the recall regarding maternal weight may have affected our study sample as the 5% weight loss was calculated based on self-reported pre-pregnancy weight and gestational weight. Indeed, self-reported pre-pregnancy weight have been shown to be under-reported [38]. Another limitation was that we relied on self-reporting of HG diagnosis. Finally, our cohort of women was self-selected, based on recruitment via social media and the patient organization for HG. Women with more negative experiences may be more motivated to share their experiences than other women with HG. Assessing representativeness of our study sample is challenging due to lack of national statistics characterizing women with HG in pregnancy. However, compared to a prior Norwegian study among women classified as having HG based on maternal self-report of hospitalization due to nausea and vomiting during pregnancy [39], we found that the participants in our study were more often primiparous. As previous studies have found that primiparous women are less willing to use prescription medications during pregnancy [40], our estimates of medication use may be an underrepresentation of the medication use among women with HG in general. Comparative multinational studies are urgently needed to assess the management and consequences of HG across Europe and worldwide. There may be reasons to believe that the negative experiences and consequences described by the women in our study could be even more profound in countries with a poorer welfare system than Norway.

## Conclusion

We found that women with HG reported suboptimal management and a lack of support from healthcare professionals, despite the severe psychosocial burden of illness and the high impact on their daily activities. Greater awareness and knowledge are needed among healthcare professionals to improve care for patients with HG. Understanding patient perspectives and acknowledging the impact of the illness are important factors for optimal HG management.

## Appendix

**Table 1:** Appendix Table 1. Sociodemographic characteristics of the study sample at the time of the interview/completion of the electronic questionnaire.

	Total population
Sociodemographic variables	*n* (%)
*Parity*^a^	
Primi parity	70 (65.4)
Multi parity	37 (34.6)
*Maternal age (years)*^*b^	
≤25	17 (17.7)
26–30	30 (31.3)
31–35	37 (38.5)
≥36	10 (10.4)
*Maternal status*^a^	
Pregnant	31 (29.0)
1st trimester	3 (2.8)
2nd trimester	19 (17.8)
3rd trimester	9 (8.4)
Not pregnant	76 (71.0)
Months since suffering of HG, median (range)	9 (0–24)
*Marital status*^b^	
Married/cohabiting/in a relationship	92 (95.8)
Divorced/single	4 (4.2)
*Employment*^b^	
Student	9 (9.9)
Employed	77 (80.2)
Homeworker	9 (9.4)
Other	1 (1.0)
*Highest level of education*^b^	
Elementary school	6 (6.3)
High school	25 (26.0)
University/college	60 (62.5)
Other	5 (5.2)
*Chronical conditions*^*b,c^	
Yes	53 (55.2)
No	40 (41.7)
*Ethnicity*^*b^	
Norwegian	83 (85.6)
Other	12 (12.3)

*Total number to not add up due to missing numbers; maternal age = 2 (2.1%), chronical conditions = 3 (3.1%), and ethnicity = 2 (2.1%).

^a^Respondents of the telephone interview, *n* = 107.

^b^Respondents of the electronic questionnaire, *n* = 96.

^c^Including allergy, asthma, diabetes 1 or 2, epilepsy, coronary heart disease, thyroid diseases, depression or anxiety, migraine, and/or other (free text entry).

**Appendix Table 2.** Ratings of health care professionals’ qualities, from the perspective of women with hyperemesis gravidarum (HG) during the interviews.

	Number of women that gave the indicated ratings, *n* (%)		
	General practitioner *n* = 107	Hospital physician *n* = 83^a^	Midwife *n* = 82^b^
*To what extent did your … have knowledge about HG?*			
Not at all	55 (51.4)	16 (20.0)	42 (51.2)
Some	13 (12.2)	12 (15.0)	15 (18.3)
Great	39 (36.5)	52 (65.0)	25 (30.5)
Unsure	0 (0.0)	3 (0.04)	0 (0.0)
*Did you feel optimally cared for and understood by your …?*			
Yes, a little	8 (7.5)	0 (0.0)	14 (17.1)
Yes, well	20 (18.7)	33 (39.8)	22 (26.8)
Yes, very well	33 (30.8)	25 (30.1)	24 (29.3)
No	46 (43.0)	25 (30.1)	22 (26.8)
*Did you trust your… to manage your HG?*			
Yes	71 (66.4)	68 (81.9)	–
No	36 (33.6)	15 (18.1)	

^a^Only 83 women were in contact with a hospital physician.

^b^Only 82 women were in contact with a midwife. No questions were asked about trust in their midwife.
